# Autonomic Neuropathy is Associated with More Densely Interconnected Cytokine Networks in People with HIV

**DOI:** 10.21203/rs.3.rs-2670770/v1

**Published:** 2023-03-17

**Authors:** Steven Lawrence, Bridget R. Mueller, Emma K. T. Benn, Seunghee Kim-Schulze, Patrick Kwon, Jessica Robinson-Papp

**Affiliations:** New York University Langone Medical Center; Icahn School of Medicine at Mount Sinai; Icahn School of Medicine at Mount Sinai; Icahn School of Medicine at Mount Sinai; New York University Langone Medical Center; Icahn School of Medicine at Mount Sinai

**Keywords:** Autonomic neuropathy, HIV, immune function, network analysis

## Abstract

**Introduction.:**

The autonomic nervous system (ANS) plays a complex role in the regulation of the immune system, with generally inhibitory effects via activation of β-adrenergic receptors on immune cells. We hypothesized that HIV-associated autonomic neuropathy (HIV-AN) would result in immune hyperresponsiveness which could be depicted using network analyses.

**Methods.:**

Forty-two adults with well-controlled HIV underwent autonomic testing to yield the Composite Autonomic Severity Score (CASS). The observed range of CASS was 2–5, consistent with normal to moderate HIV-AN. To construct the networks, participants were divided into 4 groups based on the CASS (i.e., 2, 3, 4 or 5). Forty-four blood-based immune markers were included as nodes in all networks and the connections (i.e., edges) between pairs of nodes were determined by their bivariate Spearman’s Rank Correlation Coefficient. Four centrality measures (strength, closeness, betweenness and expected influence) were calculated for each node in each network. The median value of each centrality measure across all nodes in each network was calculated as a quantitative representation of network complexity.

**Results.:**

Graphical representation of the four networks revealed greater complexity with increasing HIV-AN severity. This was confirmed by significant differences in the median value of all four centrality measures across the networks (p≤0.025 for each).

**Conclusion.:**

Among people with HIV, HIV-AN is associated with stronger and more numerous positive correlations between blood-based immune markers. Findings from this secondary analysis can be used to generate hypotheses for future studies investigating HIV-AN as a mechanism contributing to the chronic immune activation observed in HIV.

## Introduction

The autonomic nervous system (ANS) plays a key role in regulating the function of both innate and adaptive immunity.^1^ The sympathetic, parasympathetic and sensory branches of the ANS are all involved, with local effects mediated by direct sympathetic innervation of primary and secondary lymphoid organs, and modulation of systemic inflammation occurring via: 1) surveillance of blood borne immune mediators by the autonomic sensory system, and 2) the indirect effects of the parasympathetic nervous system on the spleen. Given the important effects of the ANS on the immune system, it is expected that disruption of autonomic function, for example autonomic neuropathy (AN), might cause changes in immune function, however this is currently understudied. AN is a relatively common, but often subclinical, feature of many chronic systemic diseases that strain the metabolic capacity of peripheral axons, with two common examples being diabetes mellitus and HIV.^2, 3^ Interactions between AN and the immune system may be particularly relevant in the case of people with HIV given pre-existing abnormalities in immune function some of which persist despite optimal antiretroviral treatment.^4, 5^

HIV-associated AN (HIV-AN) is generally mild to moderate in severity and characterized by partial loss of autonomic nerve fibers of different types,^3^ thus its effects on immune function might be expected to vary on the basis of which fibers were impaired. For example, given that the sympathetic branch of the ANS generally has an inhibitory effect on immune cells via the action of norepinephrine on β-adrenergic receptors,^1^ immune cells in dennervated locations might be expected to display exaggerated responsiveness or a lower threshold for response to antigenic stimuli or proinflammatory cytokines. If autonomic sensory fibers are lost, critical information regarding systemic levels of inflammatory mediators may not efficiently reach the central nervous system (CNS),^6^ blunting its modulatory capacity, which might be compounded by loss of the systemic anti-inflammatory capacity of parasympathetic fibers.^7^ Taken together these effects might lead to a loss of specificity and restraint in immune responsiveness.

Importantly, these changes might not result in the elevation of any one immune marker but rather dysfunction of the system as a whole. Prior studies have used cytokine network analyses to quantify such global immune activation in people with HIV,^8, 9^ as well as other conditions such as cancer,^10, 11^ chronic fatigue syndrome,^12^ and pulmonary arterial hypertension.^13^ Such analyses generate graphical representations of cytokine networks based on bivariate correlations between individual cytokines, and can be used to quantify and compare network characteristics between groups of patients. In general, these studies have shown denser, more interconnected networks in inflammatory disease states compared to controls, and have suggested such findings may represent a muted form of “cytokine storm.”^9^ To our knowledge, such analyses have not previously been used to study the relationship between autonomic and immune function.

Herein we describe network analyses examining the relationships between 44 blood-based immune markers in people with HIV with varying degrees of HIV-AN. We hypothesized that more severe HIV-AN would result in denser, more complex networks, reflecting a tendency toward an “all or nothing” immune response due to a loss of autonomic modulation. We also report an exploratory analysis using a machine learning algorithm to back-predict HIV-AN severity from immune marker data.

## Methods

Study overview. This is a secondary data analysis of participants recruited to two other studies of HIV-AN, (one cross-sectional observational study and one pilot clinical trial), conducted at a single academic medical center.^14, 15^ Both studies had the same inclusion/exclusion criteria and recruited from the same patient population. The baseline procedures for the clinical trial were identical to those of the single cross-sectional visit in the observational study. In the case of data collected in the clinical trial, only baseline data (i.e., prior to any intervention) were used herein. Brie y, included participants in both parent studies were adults (≥ 18 years) living with HIV and treated with combined antiretroviral therapy (CART) for at least 3 months, with HIV-1 plasma RNA load of ≤ 100 copies/ml. Confounders for autonomic dysfunction (e.g. diabetes, interfering medications) were exclusionary. All procedures were performed in accordance with a protocol approved by the Institutional Review Board of the Icahn School of Medicine at Mount Sinai (ISMMS). All participants provided written informed consent.

The Composite Autonomic Severity Score (CASS) was calculated for each participant based on the results of a standardized battery of autonomic function tests.^16^ This battery has been described previously and consists of: quantitative sudomotor axon re ex testing (QSART), heart rate response to deep breathing (HRDB), Valsalva maneuver (VM), and tilt table testing.^16, 17^ In general the CASS is scored on a scale of 0–10, with higher numbers indicating worse autonomic function. The observed range for CASS in the data was 2 to 5, consistent with normal to moderately abnormal autonomic function, thus for the purposes of this analysis participants were divided into four groups based on CASS score (i.e., score of 2, 3, 4 or 5).

Blood samples were processed to isolate the plasma within one hour of phlebotomy, stored at −80°C, and used only once (without repeated freeze thaw cycles) according to standard laboratory protocol.^14^ Plasma samples were then analyzed by our institution’s Human Immune Monitoring Center, using a bead-based ELISA method by Milliplex xMAP technology (Millipore, Billerica, MA) with a Luminex 200 system (Luminex Corporation, Austin, TX). We used a premixed 41 plex human cytokine/chemokine panel which includes: EGF, Eotaxin, G-CSF, GM-CSF, IFNα2, IFNγ, IL-10, IL-12P40, IL-12P70, IL-13, IL-15, IL-17A, IL-1RA, IL-1α, IL-1β, IL-2, IL-3, IL-4, IL-5, IL-6, IL-7, IL-8, IL-9, IP-10, MCP-1, MIP-1α, MIP-1β, RANTES, TNFα, TNFβ, VEGF,FGF-2, TGF-α, FLT-3L, Fractalkine, GRO, MCP-3, MDC, PDGF-AA, PDGF-AB/BB, sCD40L. Additional analytes were sCD14, sCD163 which are monocyte activation markers commonly used in studies of people with HIV.^18, 19^ We also measured high mobility group box-1 (HMGB-1), an inflammatory biomarker which has been studied both in the context of HIV and autonomic function.^20, 21^ Results were expressed as mean fluorescence intensity (MFI); laboratory quality assurance procedures have been described previously.^14^

Generation of Networks. All analyses and figures were generated using R statistical computing language. The package qgraph (1.9.2) was used for network and graphical analysis, and caret (6.0.93) for model building. Participants were divided into four groups based on CASS scores (i.e. score of 2, 3, 4 or 5) and a network was generated for each group with the 44 blood-based immune markers as nodes and the bivariate Spearman’s rank correlation coefficients as weighted, non-directional edges between nodes. All bivariate correlations regardless of strength or directionality were included in the creation of the network. However, including all edges in the network figures made them illegible and so a cutoff of an absolute value of 0.7 or greater was used as the criterion for inclusion of an edge in the figures. Positive correlations between nodes were depicted as blue and negative correlations as red; with wider darker edges representing stronger correlations. In the main network figures, node color was chosen based on functional groupings of the immune markers (angiogenic growth factors = red; anti-inflammatory = blue; chemokines = green; multifunctional growth factors = purple; hematopoetic growth factors = orange; interferons = yellow; monocyte activation markers = brown; pro-inflammatory = pink; type 2 inflammatory = grey).

To further illustrate network structure, we performed exploratory graphical analysis to establish communities of nodes within each network using the Walktrap algorithm which identifies communities in large networks via random walks, assigning each node to a single community. This is based on the idea that during a random walk one is more likely to become “trapped” in a highly interconnected community. The random walks are used to compute distances between nodes based on their partial correlations, and nodes are assigned into communities via bottom-up hierarchical clustering which initially assumes each node is a singleton cluster and then successively merges pairs of clusters according to the variance explained. In contrast to other methods such as eigenvector decomposition, spine glass, or label propagation, the Walktrap algorithm is sensitive to edge weights and is among the most stable for determining communities within networks.^22, 23^

Descriptions of Network and Node Characteristics. For each node within each network, four centrality measures were calculated using the centrality function in R within the qgraph package which is based on the method of Opsahl et al for weighted networks.^24, 25^ The four centrality measures were: node strength, expected influence, closeness centrality and betweenness centrality. Strength represents the sum of the absolute value of the weights of all edges connected to the node. Strength is the simplest measure of a node’s overall importance or involvement in a weighted network, reflecting especially local involvement given that it accounts only for direct (i.e., one step) connections. Expected influence is similar to node strength except that the original sign (negative or positive) of the edge weight is retained, which may help identify nodes with prominent negative associations.

Closeness and betweenness centrality are both based on the calculation of the shortest (i.e., most efficient) paths through the network to get from a given node to all other nodes. In a weighted network, the shortest path calculation considers both the number of nodes and the absolute value of edge strengths on the path (preferring fewer nodes and higher strength edges). Once all shortest paths are calculated, closeness centrality for a given node represents the average distance from the node to all other nodes traveling along the shortest paths. Betweenness centrality represents the number of shortest paths that pass through the node. A node with high betweenness centrality may be conceptualized as a “gatekeeper” connecting different parts of a network.^26^

Additional statistical analyses. While the scope of our network analysis was primarily descriptive in nature, statistical analyses were performed to evaluate interplay among the centrality measures and explore the relationship between immune and autonomic function. More Specifically, the Kruskal Wallis test was used to compare the centrality measures between networks.

Machine learning-based classification methods were applied to examine whether the immune mediator data could be used to back-predict the CASS score of individual participants. Specifically, we chose an ordinal random forest model given the ordered nature of CASS scores and the robustness of the model to differences in group sizes.^27^ Given that our overarching hypothesis was that HIV-AN would affect the relationships between immune mediators rather than their individual levels, we transformed the raw immune mediator data into z-scores, as the distance (and direction) from the mean was a stronger classifier than the raw data. We used data from 60% of the participants in a training set and the remaining 40% in the validation set modeling using repeated cross validation. A confusion matrix (i.e., a cross-tabulation of actual versus predicted CASS score) was created and used to estimate performance metrics for the model: accuracy, macro F1 score (given its robustness to imbalanced groups),^28^ sensitivity, specificity. Finally, we used variable importance to determine which immune markers contributed most to the model’s classification. Variable importance reflects the effect of variable permutation on the model’s accuracy. If the variable is unimportant, permutation of its values has little effect on model accuracy and variable importance tends toward zero; in contrast highly important variables yield a scaled value closer to 100.^27^

## Results

### Participants.

Participant characteristics are summarized in Table 1. Overall, the sample had a mean age of 57 years, was racially and ethnically diverse and about three-quarters male. Many participants had longstanding HIV infection, with a mean self-reported disease duration of 20 years, but all were currently well-controlled (as per study inclusion/exclusion criteria). Women were particularly underrepresented in the CASS = 3 group, in which only one of the 17 participants was female (p = 0.044). Otherwise, there were no significant demographic differences across the groups.

### Characteristics of the Networks.

**Figure 1:**
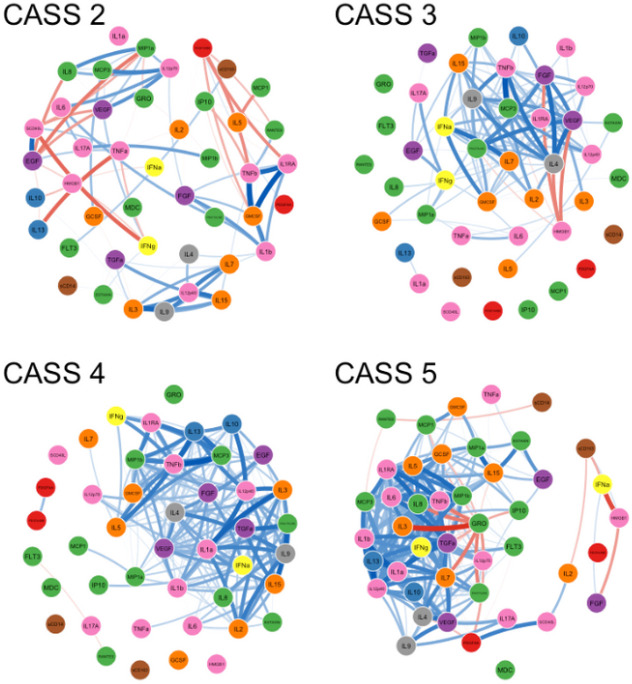
Correlation networks of immune markers. Each network represents a distinct group of participants defined by their CASS score (higher CASS reflects poorer autonomic function). Edges between nodes are all non-parametric bivariate correlations with an absolute value ≥ 0.7 (blue=positive, red=negative; weight proportional to correlation strength). Increasing network density is observed with higher CASS.

Figure 1 displays the correlation networks between immune markers for participants in each of the four CASS groups, with positive correlations in blue and negative correlations in red (only correlations with an absolute value ≥ 0.7 are shown). Increasing density of positive correlations with higher CASS scores (i.e., worse autonomic function) can be appreciated visually. Regarding negative correlations, there were a total of 22 bivariate negative correlations involving multiple nodes in the CASS = 2 network. In contrast, strong negative correlations were much more circumscribed in the other networks. Specifically, in the CASS = 3 network, a single marker, HMGB-1, was a member of all the strong bivariate negative correlations. In the CASS = 4 network there was only one strong negative bivariate correlation (RANTES to FLT3). In the CASS = 5 network, all the strong negative correlations within the main body of the network involved a single node (GRO). Thus, in contrast to the positive correlations, negative correlations tended to decrease in diversity with increasing CASS.

Network metrics are summarized in Table 2. All centrality measures differed significantly between the four networks. Strength, expected influence and closeness centrality tended to increase with CASS. In the case of strength and expected influence, this indicates that in the higher CASS networks nodes had significantly more numerous and stronger correlations with other nodes. For strength, which uses the absolute values of the correlation strength, this was a nearly linear increase, whereas for expected influence, which accounts for the correlation strength and direction, the increase occurred between the CASS = 2 and CASS = 3 networks and was then relatively at. Median closeness centrality increased linearly from CASS = 2 to CASS = 5, indicating a steady decrease in the shortest distance path between nodes overall, another reflection of more highly interconnected networks in the higher CASS networks. Betweenness centrality, which reflects how often a node is on the shortest path between other nodes, displayed a distinct pattern, being on average higher in the CASS = 2 network and lower in the other three. This is likely because the greater number of paths through the higher CASS networks decreases the likelihood that an individual node is on the shortest path between any two other nodes, or stated another way: with so many paths, fewer nodes have key bridging functions.

**Figure 2:**
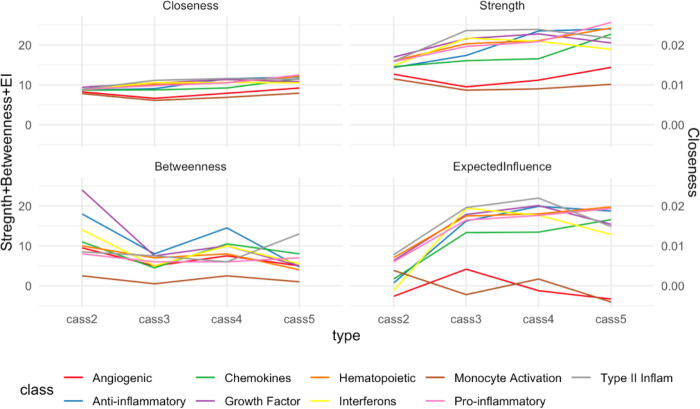
Network centrality measures. Each panel corresponds to one centrality measure (closeness and betweenness centrality, strength, expected influence). Within each panel, each colored line represents the median value of that centrality measure for a given functional class of immune markers across the four CASS groups. Similar trajectories of the lines within each panel suggests that observed differences in centrality measures between the four CASS networks were not clearly driven by any particular group of immune mediators.

Figure 2 demonstrates that in general there was no clear difference in the centrality measures between different functional classes of immune mediators. Monocyte activation markers and angiogenic growth factors (brown and red lines respectively in Fig. 2) tended to separate somewhat from the other categories, however, these were categories which held only two markers each.

### Communities within the Networks.

**Figure 3:**
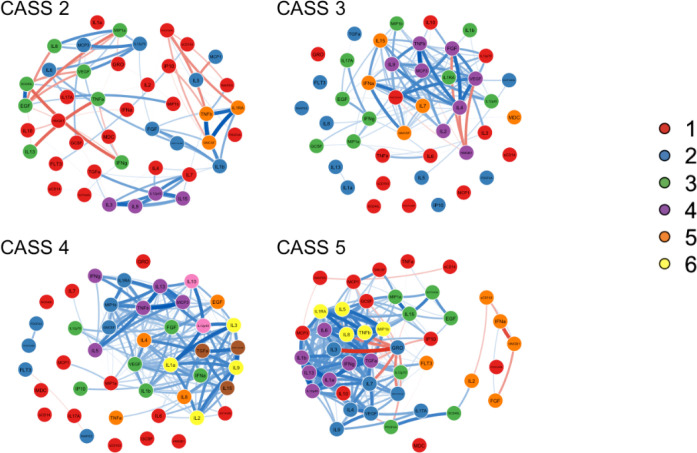
Communities within the networks. This gure replicates Figure 2, except that the node color is corresponds to community membership as determined by the Walktrap algorithm. In the CASS=2 network complex positive and negative relationships within and between communities are seen. Whereas in the other CASS networks communities are primarily defined by strong positive correlations between nodes.

The Walktrap algorithm identified between five and eight communities within each network as depicted in Fig. 3, in which the node colors have changed to reflect community but otherwise the networks remain the same as Fig. 2. Given the “bottom-up” approach of the algorithm, the first community in each network (community #1, shown in red) is typically the largest and most heterogenous and generally contains the nodes with lower centrality measures, whereas communities #2 through #8 are generally progressively smaller and more insular. The CASS = 2 and CASS = 3 networks had five communities each, whereas the CASS = 4 network had eight communities and the CASS = 5 community had six.

Examination of the communities within the CASS = 2 network suggests several elements of structure which were not present in the other networks. For example, community #5 (orange) was comprised of three nodes (TNF-β, GMCSF and IL-1 receptor antagonist (IL-1RA)) with: 1) very strong positive correlations with one another (rho > 0.9 for all); 2) strong positive connections to nodes in community #2 (blue) with high positive expected influence (FGF and IL-1b); and 3) strong negative connections to nodes in community #1 (red) with high negative expected influence (IP10 and PDGFAABB). These connections resulted in a relatively high expected influence for community #5 (median = 6.0, compared with 3.8 for the network overall). Community #4 (purple) was also characterized by strong correlations between its four members (IL-3, IL-9, IL12p40 and IL-15) and high expected influence (median = 9.1) based in part on strong positive correlations with the three highest expected influence nodes in network #1 (TGF-α, IL-4 and IL-7, median expected influence = 8.0). Community #3 (green) has two subgroups. One subgroup is comprised of EGF, VEGF and sCD40L which are all strongly positively correlated with one another and tend to be negatively correlated with the remaining members of community #3. Community #2 (blue) has one negatively correlated couplet (MCP-1 and IL-5) which is relatively separate from the other community members which are connected by multiple positive correlations.

In contrast to the complex community relationships in the CASS = 2 network, in the other CASS networks most of the communities are defined by particularly strong positive connections between multiple nodes, although there are a few notable exceptions. In the CASS = 3 network, community #4 (purple) contains HMGB-1 and several nodes with which it has strong negative connections. Similarly, in the CASS = 5 network, community #2 (blue) contains some strong negative correlations between GRO and other community members, and community #5 (orange) is characterized mostly by negative correlations with HMGB-1 and IFN-α at its center

### Predicting CASS group membership from immune mediator data.

The ordinal random forest model had 73% accuracy, indicating that it correctly assigned 73% of participants overall (i.e., across all CASS scores) to their correct CASS group. The model’s macro F1 score (an alternative performance metric which accounts for imbalance in the number of participants in each CASS group) was 72%, similar to accuracy. However, the model’s predictive value was not significantly different (p > 0.05) from the no information rate (i.e., the rate of correct classification achievable by chance alone based on the known distribution of CASS scores). Sensitivity and specificity of the model by CASS score is summarized in Table 3. As shown, the model correctly classified all participants with a CASS of 2, 4, or 5, but incorrectly classified 40% of participants with a CASS of 3. Examination of variable importance revealed that the top five immune markers contributing to the predictive value of the model (variable importance factors of > 75 for each) were: TGF-α, MDC, IL-7, IL12p40, and IL-15.

## Discussion

In this study, we used network analyses to depict and describe differences in relationships between immune markers in people living with HIV with normal autonomic function and varying degrees of HIV-AN. We found that, overall, positive correlations between immune markers increased with worsening autonomic function, reflected by increases in median centrality measures in the higher CASS networks. Similarly, strong negative correlations between immune markers decreased in their strength and diversity with increasing CASS. Given the cross-sectional, associational nature of these data and the relatively small sample size, this work cannot be used to establish causative mechanisms, but can be used to generate hypotheses regarding the possible effects of HIV-AN on immune networks.

We hypothesize that at least some of the observed correlations between immune markers are reflections of regulatory feedback loops. Both positive and negative feedback loops are key features of biologic systems. In general, negative feedback loops are appropriate for providing stability or maintenance of the status quo. In contrast, positive feedback loops are useful for rapidly amplifying a physiologic process in response to a threat, and an external control is typically required to terminate the response. Given that a main function of the immune system is to respond quickly and effectively to perturbations, such as infection or tissue damage, one would expect positive feedback loops to be prominent in its structure, and indeed this is the case. For example, in the innate immune system, activated neutrophils can directly enhance their own activity and movement toward a target via autocrine and paracrine signaling, including the release of certain chemokines.^29^ The process of T-cell activation provides an example of a positive feedback loop within the adaptive immune system. Dendritic cells present antigen to T-cells and also provide costimulatory signaling to activate the T-cell. The activated T-cell then provides positive feedback to the dendritic cell resulting in increased expression of costimulatory signaling molecules.^30^

Given that chronic HIV is a proinflammatory state, and inflammation is characterized by positive feedback loops, our networks suggest the hypothesis that the ANS may be an important external interrupter of inflammatory positive feedback loops. Specifically, in the CASS = 2 network, the presence of multiple negative correlations dispersed throughout the network could reflect underlying negative feedback loops mediated by a properly functioning ANS, which are then lost in the higher CASS networks. Pre-clinical models suggest anatomic and physiologic bases for such a phenomenon. Anatomically, under normal circumstances the sympathetic nervous system (SNS) has the potential to regulate the immune system in a nuanced and site specific manner, given that SNS fibers are a pervasive presence in all lymphoid organs.^1^ SNS fibers also have the ability to respond directly to certain pathogens via activation of toll-like receptors (TLRs).^31^ Moreover it has been hypothesized that SNS fibers innervating secondary lymphoid organs may display neuroplasticity, for example, retracting from the spleen in order to disinhibit an appropriate inflammatory response, and then later returning to curtail it.^32^ In addition to these local mechanisms, the SNS can also exert systemic effects via the sympatho-adrenal-medullary (SAM) axis through which the SNS stimulates the adrenal medulla to release catecholamines directly into the systemic circulation.^33^

In addition to this neuroanatomy, a significant body of research documents the physiologic response of diverse immune cells to norepinephrine (NE), the main neurotransmitter of the SNS. Immune cell response to NE, as recently and comprehensively reviewed,^31^ is permitted by the presence of β- and to a lesser extent α-adrenergic receptors (β-AR and α-AR respectively) on both innate and adaptive immune cells. In general, β-ARs are lower affinity but more numerous, and so NE at the relatively high concentrations supplied by local SNS terminals results in greater activation of β-AR which has a generally suppressive effect. In contrast, α-ARs which have a generally pro-inflammatory effect, are less numerous but higher affinity and so may be preferentially activated by diffuse low level catecholamines, such as is released via the SAM axis (e.g. in response to chronic stress).^34^

The sensory and parasympathetic branches of the ANS (both contained predominantly within the vagus nerves) are also important regulators of immune function, despite their apparent lack of significant direct innervation of lymphoid organs.^1^ Vagal sensory fibers detect the presence of cytokines in the circulation, and convey this information to the CNS with cytokine-specific ring patterns referred to as “neurograms.”^6^ Moreover, blocking of vagal sensory fibers lessens CNS-mediated responses to infection including fever and sickness behavior.^35^ The effect of the parasympathetic on immune function has also been well described and is sometimes referred to as the cholinergic anti-inflammatory pathway, given that acetylcholine (ACh) is its main neurotransmitter.^7^ Although certain portions of the pathway remain uncertain, it is clear that activation of vagal parasympathetic nerve fibers ultimately inhibits the release of inflammatory mediators (e.g. IL-6 and TNF-α) into the circulation from splenic macrophages in response to a systemic inflammatory stimulus such as lipopolysaccharide (LPS).^36^ Treated HIV has been conceptualized as a state of chronic low-level exposure to antigenic stimuli such as LPS due to alteration of the gut microbiome and increased translocation across a compromised gut barrier.^37^ Thus it is possible that partial loss of sympathetic, parasympathetic, and sensory autonomic fibers in HIV-AN leads to a decreased ability to control the intensity and breadth of immune responsiveness in people living with HIV. Indeed, prior work has shown more closely correlated cytokine networks in people living with HIV compared to uninfected controls,^8, 9^ although not in the context of autonomic dysfunction.

This study has important limitations. The sample size is small, and this is a secondary data analysis of data collected from a single academic center and so the findings require replication in a larger cohort in which such a network analysis is a pre-planned primary outcome. Another limitation is that machine learning models are better suited to larger sample sizes, although among such models, ordinal random forests have been proposed as more adaptable for smaller sample sizes. We have previously shown that HIV-AN is associated with increased burden of medical comorbidities,^38^ and so it is possible that the observed increases in network density at higher CASS scores are not due to the autonomic dysfunction but rather due to other medical issues. Moreover, women and men were not evenly distributed between the four CASS groups and so it is possible that differences in immune function could be related to sex-based differences. It is a limitation of this network analysis approach that such potential confounders cannot be readily controlled for.

Despite these limitations this study provides preliminary evidence supporting a potential role of HIV-AN in the state of chronic immune activation observed in people living with HIV. Future work is needed to replicate the findings in larger cohorts, and to employ methodology which will enable more precise mechanistic characterization of autonomic-immune interactions. These may include combinations of serum catecholamine measurements, “whole body” imaging of sympathetic innervation with radiotracers, detailed immune cell phenotyping, and advanced computational methods to identify phenotypes of autonomic and immune function and their intersections.

## Figures and Tables

**Table 1:** Participant characteristics.

	Overall (n=42)	CASS = 2 (n = 8)	CASS = 3 (n = 17)	CASS = 4 (n = 10)	CASS = 5 (n = 7)	p-value

Age, mean (SD)	57.2 (6.5)	60 (4.8)	57 (8.0)	55 (4.6)	59 (5.5)	0.284

Sex, n (% of column)						
Male	31 (74%)	6 (75%)	16 (94%)	6 (60%)	3 (42%)	0.044
Female	11 (26%)	2 (25%)	1 (6%)	4 (40%)	4 (57%)	

Race						
Black	23 (55%)	5 (63%)	8 (47%)	6 (60%)	4 (57%)	0.925
White	18 (43%)	3 (38%)	8 (47%)	4 (40%)	3 (42%)	
Other	1 (3%)	0 (0%)	1 (6%)	0 (0%)	0 (0%)	

Ethnicity						
Hispanic/Latinx	12 (29%)	4 (50%)	3 (18%)	3 (30%)	2 (29%)	0.423
Non-Hispanic/Latinx	30 (71%)	4 (50%)	14 (82%)	7 (70%)	5 (71%)	

Current CD4 + T-cell count, mean (SD)	693 (330)	607 (165)	591 (353)	899 (319)	746 (336)	0.096

Nadir CD4 + T-cell count, mean (SD)	297 (260)	208 (203)	257 (274)	374 (265)	366 (286)	0.506

Years of HIV infection, mean (SD)	20 (7.4)	20 (7.1)	19 (9.2)	22 (5.3)	22 (5.9)	0.670

**Table 2:** Network metrics

	CASS = 2	CASS = 3	CASS = 4	CASS = 5	p-value
Node strength	15.0 (13.7, 16.2)	19.6 (14.4, 21.9)	20.9 (14.5, 23.7)	23.5 (17.0, 25.7)	<0.001
Expected influence	3.8 (0.4, 7.5)	16.8 (10.8, 18.7)	18.2 (10.7, 20.9)	16.9 (5.3, 19.8)	<0.001
Closeness centrality	0.009 (0.008, 0.009)	0.010 (0.008, 0.011)	0.011 (0.009, 0.012)	0.012 (0.010, 0.013)	<0.001
Betweenness centrality	10.5 (6.0, 14.0)	5.0 (1.0, 14.0)	7.5 (3.0, 12.0)	5.0 (2.0, 10.25)	0.025

**Table 3:** Sensitivity and Specificity of the Ordinal Random Forest Model

	CASS = 2	CASS = 3	CASS = 4	CASS = 5
Sensitivity	100%	60%	100%	100%
Specificity	92%	100%	85%	93%
